# Mitochondrial Ca^2 +^ Is Related to Mitochondrial Activity and Dynamic Events in Mouse Oocytes

**DOI:** 10.3389/fcell.2020.585932

**Published:** 2020-10-27

**Authors:** Feng Wang, Tie-Gang Meng, Jian Li, Yi Hou, Shi-Ming Luo, Heide Schatten, Qing-Yuan Sun, Xiang-Hong Ou

**Affiliations:** ^1^Fertility Preservation Lab, Reproductive Medicine Center, Guangdong Second Provincial General Hospital, Guangzhou, China; ^2^State Key Laboratory of Stem Cell and Reproductive Biology, Institute of Zoology, Chinese Academy of Sciences, Beijing, China; ^3^Department of Veterinary Pathobiology, University of Missouri, Columbia, MO, United States

**Keywords:** mitochondrial Ca^2+^, oocyte activation, oocyte maturation, oocyte metabolism, Ca^2+^ oscillations

## Abstract

Mitochondrial energy insufficiency is strongly associated with oocyte activation disorders. Ca^2+^, especially that in the mitochondrial matrix, plays a pivotal role in mitochondrial energy supplementation, but the underlying mechanisms are still only poorly understood. An encoded mitochondrial matrix Ca^2+^ probe (Mt-GCaMP6s) was introduced to observe mitochondrial Ca^2+^ ([Ca^2+^]_m_) dynamic changes during oocyte maturation and activation. We found that active mitochondria surrounding the nucleus showed a higher [Ca^2+^]_m_ than those distributed in the cortex during oocyte maturation. During oocyte partheno-activation, the patterns of Ca^2+^ dynamic changes were synchronous in the cytoplasm and mitochondria. Such higher concentration of mitochondrial matrix Ca^2+^ was closely related to the distribution of mitochondrial calcium uptake (MICU) protein. We further showed that higher [Ca^2+^]_m_ mitochondria around the chromosomes in oocytes might have a potential role in stimulating mitochondrial energy for calmodulin-responsive oocyte spindle formation, while synchronizing Ca^2+^ functions in the cytoplasm and nuclear area are important for oocyte activation.

## Introduction

According to the WHO 2016 report, at least one of 10 couples in developed countries cannot have children within 5 years of marriage, half of which are due to female infertility (Chow et al., 2016). Oocyte activation inefficiency is a major problem causing female infertility (Schatten et al., 2014). Oocyte activation rates in obese, diabetic and aging women are low, affecting the development of preimplantation embryos and also pregnancy (Minge et al., 2008; Igosheva et al., 2010; Jungheim et al., 2010; Shankar et al., 2011; Luzzo et al., 2012; Wang et al., 2017). Such disorders are strongly related with mitochondrial energy supply insufficiency (Gesink Law et al., 2007; Rittenberg et al., 2011). Resolving the low quality of oocytes caused by metabolic abnormalities and improving the efficiency of assisted reproduction are key issues that researchers need to address.

Ca^2+^ is one of the major signaling molecules regulating several cell functions, such as celldivision, arrest, and apoptosis. At fertilization, oocyte activation begins with a series of crucial steps triggered by periodical repetitive increases and decreases in intracellular Ca^2+^ ([Ca^2+^]_i_) concentrations known as [Ca^2+^]_i_ oscillations (Stricker, 1999; Wakai et al., 2013). Mitochondrial function and intracellular [Ca^2+^]_i_ are two critical factors for the oocyte’s developmental potential (Wacquier et al., 2019). Although [Ca^2+^]_i_ oscillations are required for oocyte activation (Yazawa et al., 2007), very little is known about how cytoplasmic and mitochondrial Ca^2+^ regulates the energy supply mode transition during oocyte activation.

Mitochondrial Ca^2+^ is a key factor initiating mitochondrial metabolism and regulating mitochondrial activity. Mitochondrial matrix Ca^2+^ homeostasis is strictly regulated by the transmission of Ca^2+^ between the cytoplasm, ER and mitochondria (Dumollard et al., 2006), which plays important roles in cellular physiology. Mitochondrial Ca^2+^ regulates membrane potential, stimulates rate-limiting enzymes in the Krebs cycle, and accelerates ATP synthesis (Chacko and Eliceiri, 2018). Ca^2+^ oscillations triggered by sperm-oocyte fusion also affect fertilized oocyte mitochondria and promote the transition of mitochondria from the resting state to the activation state (Dumollard et al., 2006). Our previous results showed that mitochondrial activity plays a critically important role in maintaining [Ca^2+^]_i_ oscillations (Wang et al., 2018). Although there are some reports on the mechanisms of Ca^2+^ and mitochondria interactions during cell differentiation (Mallilankaraman et al., 2012a,b), the relationship between [Ca^2+^]_m_ and mitochondrial metabolism is unknown in oocytes.

In this study, we observed the dynamic [Ca^2+^]_m_ changes during *in vitro* maturation and activation of oocytes, and we further studied the functions of matrix Ca^2+^ on mitochondrial activity, with the ultimate goal to improve oocyte quality by modifying the metabolism of mitochondria within the oocyte.

## Materials and Methods

### Ethics Statement

Female ICR mice were purchased from the Beijing Vital River Laboratory Animal Technology Co., Ltd. All mice were handled in accordance with the institutional animal care policies of the Institute of Zoology, Chinese Academy of Sciences. Mice were maintained under a 12 h light and 12 h darkness cycle in a specific pathogen-free stage at the Central Animal Laboratory of the Institute of Zoology. The Laboratory Animal Care and Use Committee of the Institute of Zoology approved this study.

### Oocyte Collection and Partheno-Activation

Female mice were injected with 10 IU pregnant mare serum gonadotropin (PMSG, Ningbo Hormone Product Co., Ltd., Cixi, China). After 44–48 h, GV oocytes were collected by ovarian mincing. Female mice were injected with 10 IU PMSG followed 44–48 h later by injection of 10 IU human chorionic gonadotropin (hCG, Ningbo Hormone Product Co., Ltd., Cixi, China). After 15 h of hCG, ovulated MII oocytes were collected and denuded in 1 mg/mL hyaluronidase. Matured oocyte [Ca^2+^]_i_/[Ca^2+^]_m_ oscillations were induced by 10 mM strontium chloride (SrCl_2_, 10025-70-4, Sangon Biotech, Shanghai, China) in Ca^2+^-free CZB for 4 h. After partheno-activation, eggs were transferred into KSOM (MR-106, Merck Millipore, MA, United States).

### Plasmid Construction of Vectors

An encoded Ca^2+^-sensitive probe was constructed based on the cpGFP (Circularly Permuted Green Fluorescent Protein) system and calmodulin (GCaMP6s) to detect Ca^2+^ concentrations (Akerboom et al., 2012; Zhong and Schleifenbaum, 2019). The mitochondrial location probe plasmid was constructed to express the Ca^2+^ detection protein probe with mitochondrial localization signal peptide cloned from Trmt10c gene (Metodiev et al., 2016; Oerum et al., 2018). For cloning of genes encoding MICU protein, voltage-dependent anion selective channel (VDAC) and CaMs, The coding sequences (CDS) were obtained by RT-PCR with template of ovary cDNA cloned into the pMD-18-T vector (Takara, Dalian, Liaoning, China). 18-T vector were sliced and linked CDS to upstream of mCherry. Two plasmids, p-UC57-Mt-GCaMG6s (Supplementary Figure S1A) and p-GEMHE-MICU-mCherry (Supplementary Figure S1B), were dissolved in water and stored at −20°C.

### *In vitro* Transcription of cRNA

Templates of *in vitro* transcription from constructed plasmids were obtained by PCR with primers M13F and M13R (Supplementary Figures S1C–F). PCR products were diluted in RNAse-free water. cRNA transcripts were synthesized *in vitro* with T7 RNA polymerase mMESSAGE mMACHINE T7 kit (Ambion, Life Co., Calsbad, CA, United States). Poly(A) tail was added to the sequence end by polymerase tailing kit (PAP5104, Lucigen, Beijing, China). The RNA solutions were then stored at −80°C in a final concentration of 400 μg/mL until further use. Approximately 50 pl of RNA solution was injected into each GV oocyte.

### GV Oocyte Microinjection

The M2 medium and M16 medium containing 2.5 μM milrinone were prepared and warmed to 37°C. Milrinone is a phosphodiesterase inhibitor that maintains meiotic arrest once oocytes are removed from the follicles (Stein and Schindler, 2011). Micro-drops each containing 20 μl M16 medium with milrinone were prepared in a dish and overlaid with mineral oil. Injection pipettes were made by pulling borosilicate-glass capillary with filament in a mechanical puller. We used a Flaming-Brown micropipette puller (Model P-97) with the following settings: *P* = 540, Heat = 300, Pull = 130, Vel = 100, Time = 150. Microinjection platform was prepared by placing a 10 μl micro-drop of M2 medium with milrinone on a chamber slide, and the drop was covered with mineral oil and placed on the microscope stage. Injection and holding pipettes were placed into the drop of M2 medium with milrinone. Microinjection of cRNA was performed with Narishige micromanipulators (Narishige Inc., Sea Cliff, NY) under a Nikon TE 200 (Nikon UK Ltd., Kingston upon Thames, Surrey, United Kingdom) and finished within 30 min. After cRNA injection, oocytes were arrested at the GV stage in M16 medium containing 2.5 μM milrinone for 4 h. Then the oocytes were transferred to M16 medium and cultured under mineral oil at 37°, in an atmosphere of 5% CO_2_ in air for 14 h *in vitro* maturation.

### Real-Time Recording of [Ca^2+^]_i_ and [Ca^2+^]_m_ Changes

We set out to confirm [Ca^2+^]_i_ oscillations regulation on mitochondrial activity during oocyte activation. [Ca^2+^]_i_ oscillations were observed by staining with 2μM Fluo4 AM (488 nm excitation, 525 nm emission) in the partheno-activation system. Rhod2 is a classical dye for determining mitochondrial Ca^2+^ concentration ([Ca^2+^]_m_) with a higher Ca^2+^ affinity (Garcia-Perez et al., 2008; Eisner et al., 2010). We recorded [Ca^2+^]_m_ dynamic changes with 2 μM Rhod2 AM (561 nm excitation, 590 nm emission) in a partheno-activation system. Mt-GCaMP6s was introduced to obtain the patterns of mitochondrial matrix Ca^2+^ dynamic changes. Real-time images were obtained using a time-lapse confocal laser microscope (UltraVIEW-VoX; PerkinElmer, MA, United States) and recorded at 2 frames per minute. [Ca^2+^]_i_ and [Ca^2+^]_m_ intensities were detected using an argon laser.

### Real-Time Recording of Distribution of Activated Mitochondria

Activated mitochondria were imaged with 0.1 μM MitoRed (C2005, Beyotime, China), a cell permeable potential-sensitive fluorescent mitochondrial dye emitting in red (561 nm excitation, 590 nm emission) channel, which has a high affinity with higher potential mitochondria (Chazotte, 2011), by using a time-lapse confocal laser microscope (UltraVIEW-VoX; PerkinElmer, United States). The GV oocyte maturation was conducted by incubation in M16 (M7167, Sigma-Aldrich, United States) with 0.1 μM MitoRed. MII oocytes were activated in Ca^2+^-free-CZB with SrCl_2_, staining with 0.1 μM MitoRed. A software Volocity was used to analyze fluorescence intensity.

### Statistical Analysis

All experiments were conducted at least three times. No fewer than 40 oocytes were collected and examined in each experiment and no fewer than three biological repeats for one group were conducted. We presented information of samples with Means and Standard Deviations (SD). Results were analyzed by SPSS 19.0. The significance of differences among groups was analyzed by the Chi-square test; *p* < 0.05 were considered statistically significant.

## Results

### Dynamic Changes in Mitochondrial Matrix Ca^2+^ During Partheno-Activation of Oocytes

**FIGURE 1:**
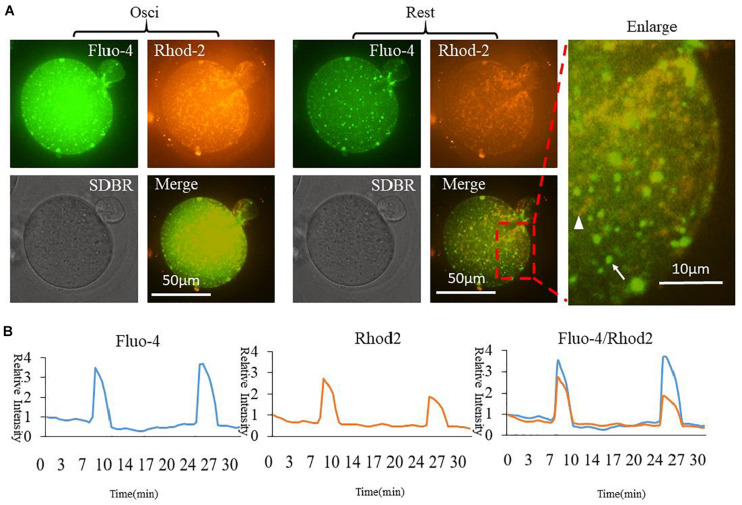
Fluo4 and Rhod2 dynamic changes during oocyte partheno-activation. **(A)** Fluo4AM and Rhod2AM staining of oscillating (Osci) and resting oocytes during activation. In the enlarged panel, arrow indicates Fluo4 staining of cytoplasmic Ca^2+^, and arrowhead indicates Rhod2 staining of mitochondrial Ca^2+^. The two dyes display clear different locations. **(B)** Relative fluorescence intensity of two continuous [Ca^2+^]_i_ and [Ca^2+^]_m_ oscillations. Ordinate is marked as Relative Intensity (relative to the fluorescence intensity of the start point).

In order to compare cytoplasmic and mitochondrial Ca^2+^ dynamic changes during oocyte partheno-activation, three dyes were employed to observe the Ca^2+^ concentration patterns in PMSG/HCG primed ovulated MII oocytes. Firstly, we detected Ca^2+^ dynamic changes in the cytoplasm ([Ca^2+^]_i_) and mitochondria ([Ca^2+^]_m_) by co-staining with Fluo4 AM (Figure 1A arrow) and Rhod2 AM (Figure 1A arrowhead). Oocyte [Ca^2+^]_i_ oscillations were detected using Fluo4 AM in the partheno-activation system, while Rhod2 AM was reported as a classic dye for determining [Ca^2+^]_m_ in somatic cells (Garcia-Perez et al., 2008; Eisner et al., 2010). Rhod2 signal showed the same pattern as [Ca^2+^]_i_ oscillations (Figure 1B). Ca^2+^ concentrations in the cytoplasm and mitochondria changed synchronously during partheno-activation. We suggest that patterns of mitochondrial Ca^2+^ during oocyte activation may be the same as that of [Ca^2+^]_i_ oscillations.

**FIGURE 2:**
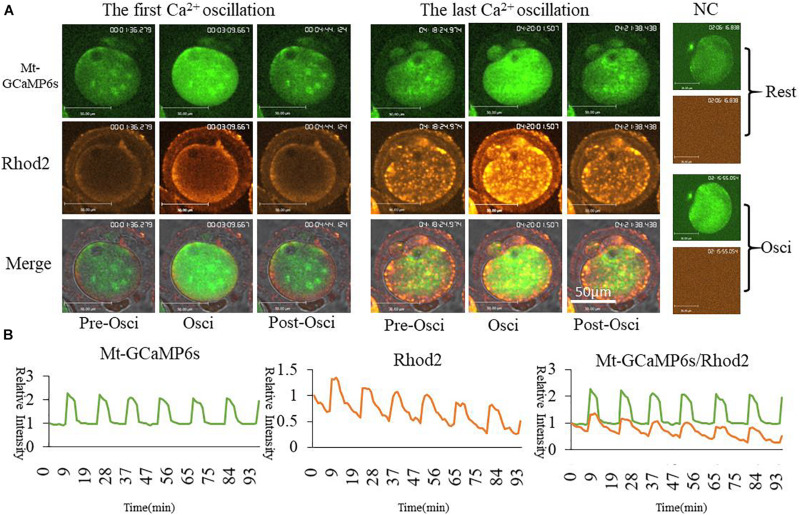
Mt-GCaMP6s and Rhod2 dynamic changes during oocyte partheno-activation. **(A)** First and last [Ca^2+^]_m_ oscillations in partheno-activated oocytes. **(B)** Relative fluorescence intensity of [Ca^2+^]_m_ oscillations as revealed by Mt-GCaMP6s and Rhod2. Patterns of Mt-GCaMP6s and Rhod2 are synchronous. Ordinate is marked as Relative Intensity (relative to the fluorescence intensity of the start point).

In order to further confirm the changes of [Ca^2+^]_m_ during oocyte activation, we applied *in vitro* transcription and microinjection (Stein and Schindler, 2011)of cRNA encoding a cpGFP (circularly permuted green fluorescent protein) based-Ca^2+^ probe with a mitochondrial signal peptide (Mt-GCaMP6s) (Supplementary Figure S1A; Akerboom et al., 2012) to determine [Ca^2+^]_m_ patterns. Indeed, most of the mitochondria could be stained by Rhod2 while only some energized mitochondria were stained by GCaMP6s (Figure 2). In comparison, GCaMP6s is more suitable for detecting the dynamic changes of [Ca^2+^]_m_ in oocytes. Finally regardless of which dye was used, [Ca^2+^]_i_ and [Ca^2+^]_m_ oscillations had the same pattern during oocyte activation. Ca^2+^ changes in the cytoplasm and mitochondria were synchronous.

### Dynamic Changes in [Ca^2+^]_m_ During Oocyte Meiotic Maturation

**FIGURE 3:**
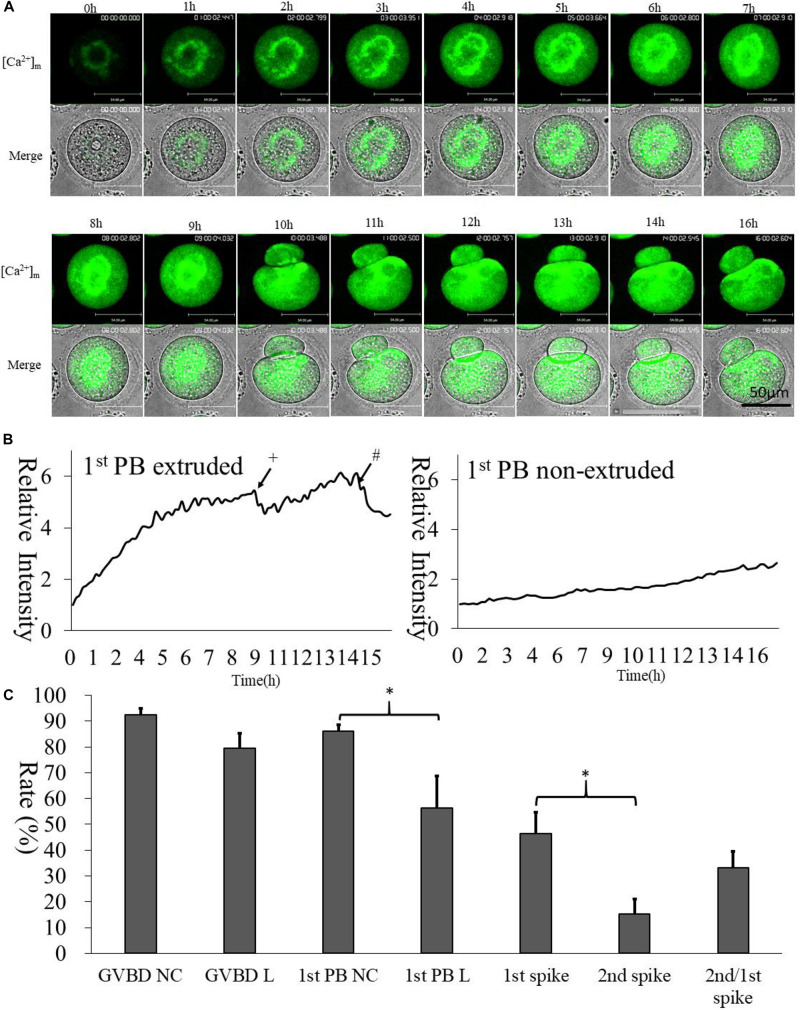
[Ca^2+^]_m_ dynamic changes during oocyte maturation. **(A)** Mt-GCaMP6s staining of GV oocyte maturation. **(B)** Fluorescence intensity of [Ca^2+^]_m_ changes in mitochondria during maturation. The left curve indicates 1st polar body-extruded oocytes, and the right curve indicates GVBD but 1st poly body- non-extruded oocytes, which lacks [Ca^2+^]_m_ dynamic changes as *in vitro* matured MII oocytes. ^+^The first polar body extrusion. ^#^The second [Ca^2+^]_m_ spike in MII oocyte. Ordinate is marked as Relative Intensity (relative to the fluorescence intensity of the start point). **(C)** Developmental rate of oocytes. “NC” indicates cRNA injected control oocytes cultured in 5% CO_2_ incubator. “L” indicated long-term laser irradiation in living cell station. *Indicates significance of differences (*p* < 0.05).

We selected Mt-GCaMP6s to observe the dynamic pattern of [Ca^2+^]_m_ changes in mitochondria during GV oocyte *in vitro* maturation (Figure 3 and Supplementary Video S1). Mitochondrial [Ca^2+^]_m_ was located to the surrounding area of the germinal vesicle, which was clearly higher than that located at the oocyte cortex. After GVBD, higher [Ca^2+^]_m_ mitochondria were observed to distribute around the spindle. Mitochondrial metabolism is much stronger in the energy-demanding part of the oocyte. Interestingly, there were two increasing [Ca^2+^]_m_ spikes during oocyte maturation (Figure 3B). The first [Ca^2+^]_m_ spike was observed at 9 h, before the first polar body extrusion, in about half of the GV oocytes examined (46.3 ± 8.3%). 1st polar body non-extruded oocytes did not show any Ca^2+^ spike. The second spike occurred 4–5 h after the first polar body emission, i.e., at about 14 h of culture in 33.2 ± 6.2% of oocytes which exhibited the first spike (Figure 3C). This coincided with the suitable window when the oocytes are ready for activation. These two time points were likely most critical for energy requirements.

### Higher [Ca^2+^]_m_ and Activated Mitochondrial Distribution in Oocytes

**FIGURE 4:**
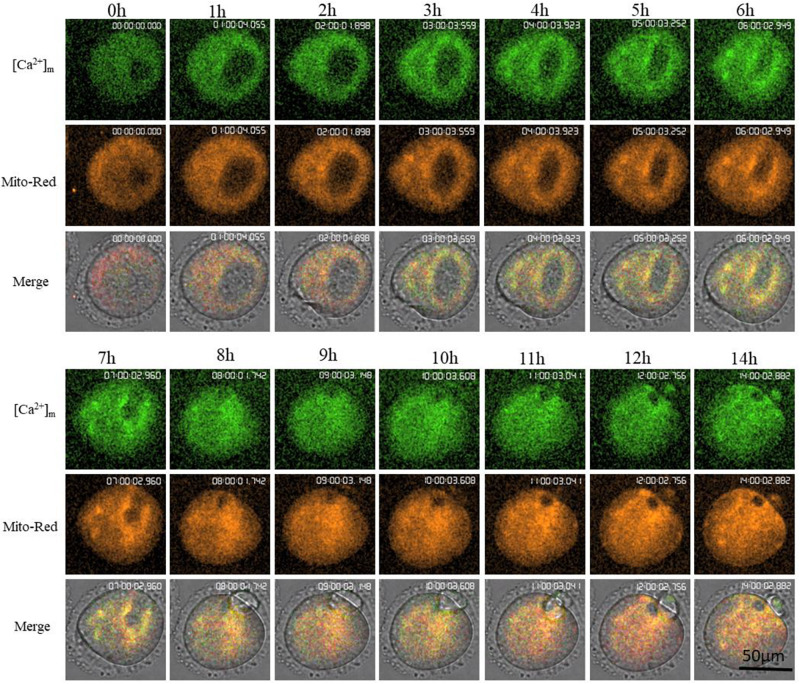
Higher [Ca^2+^]_m_ and activated mitochondria distribution during oocyte maturation. [Ca^2+^]_m_ indicates Mt-GCaMP6s staining of mitochondria. Mito-Red indicates activated mitochondria. [Ca^2+^]_m_ and Mito-Red shows co-localization.

**FIGURE 5:**
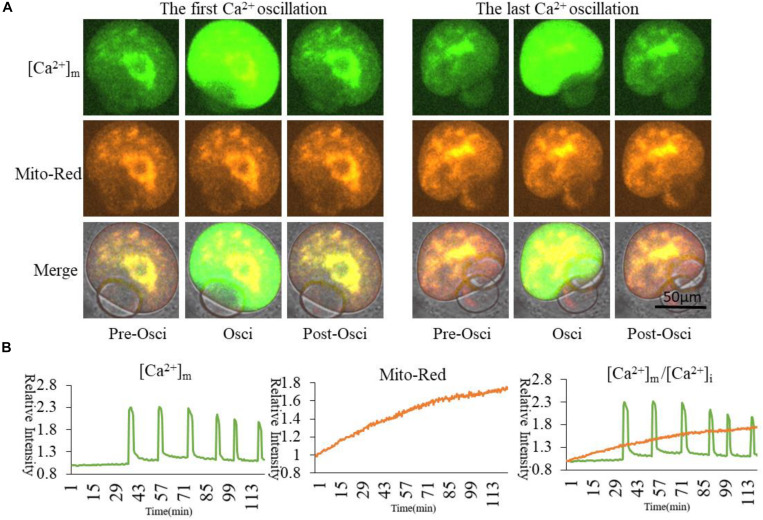
[Ca^2+^]_m_ and activated mitochondria distribution during oocyte partheno-activation. [Ca^2+^]_m_ indicates Mt-GCaMP6s staining of mitochondria. **(A)** [Ca^2+^]_m_ and activated mitochondria distribution at first and last Ca^2+^ oscillations. Mito-Red indicates activated mitochondria. One part of higher [Ca^2+^]_m_ and Mito-Red shows an evident co-localization that is maintained for a long time in mitochondria surrounding chromosomes. [Ca^2+^]_m_ in most cortical mitochondria changed with [Ca^2+^]_i_ oscillations. **(B)** Fluorescence intensity of [Ca^2+^]_m_ and Mito-Red changes during oocyte maturation and activation. Ordinate is marked as Relative Fluorescence Intensity (relative to the fluorescence intensity of the start point).

Mt-GCaMP6s was further applied to observe the dynamic relationship between [Ca^2+^]_m_ and activated mitochondrial distribution during oocyte maturation (Figure 4) and during a 4-h short term observation (Supplementary Figure S2). MitoRed-stained activated mitochondria surrounded the chromosomes and spindle with a higher [Ca^2+^]_m_. Next, we employed a partheno-activation system (Ca^2+^-free CZB with SrCl_2_) to observe the changes of [Ca^2+^]_m_ and activated mitochondria distribution during oocyte activation (Figure 5). We found that there were two types of [Ca^2+^]_m_ distributions of different mitochondria. In one part staining was maintained for a long time. The higher [Ca^2+^]_m_ co-localized with the MitoRed positive mitochondria. [Ca^2+^]_m_ in the other part of mitochondria changed as [Ca^2+^]_i_ oscillation patterns rapidly increased and decreased. We found that during activation, [Ca^2+^]_i_ oscillations in oocytes will drive Ca^2+^ changes in the mitochondria, but it does not affect these MitoRed positive mitochondria.

### Higher [Ca^2+^]_m_ Related to the Distribution of MICU

**FIGURE 6:**
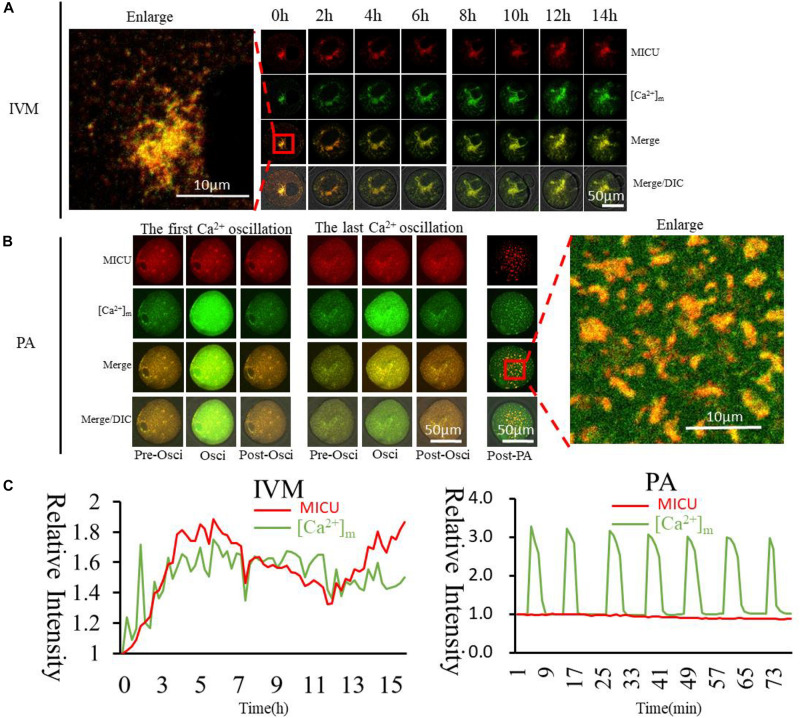
Dynamic distribution of MICU and higher [Ca^2+^]_m_ mitochondria during oocyte maturation and activation. **(A,B)** MICU and higher [Ca^2+^]_m_ mitochondria distribution during oocyte maturation and activation. IVM, *In vitro* Maturation; PA, Parthenogenetic Activation. **(C)** Fluorescence intensity of MICU (Red) and [Ca^2+^]_m_ (Green)changes during maturation and activation. Ordinate is marked as Relative Fluorescence Intensity (relative to the fluorescence intensity of the start point).

Mitochondrial calcium uniporter (MCU) has multiple subunits, and its Ca^2+^ influx activity is controlled by regulatory subunits, including mitochondrial calcium uptakers (MICU) (Xing et al., 2019). *In vitro* transcription vector of MICU was constructed to confirm the relationship between [Ca^2+^]_m_ and MICU. MICU was found to co-distribute with higher [Ca^2+^]_m_ mitochondria in the GV oocytes (Figure 6). In PA oocytes, such distribution was similar as that of activated mitochondria and higher [Ca^2+^]_m_ mitochondria. During activation, [Ca^2+^]_i_ oscillations will drive Ca^2+^ changes in the mitochondria, but it did not affect these Mito-Red positive mitochondria. MICU distribution in oocytes was similar to Mito-Red positive activated mitochondria. This may indicate that MICU is related to mitochondrial matrix Ca^2+^ and mitochondrial activity.

VDACs (voltage-dependent anion channels) are a widespread on the outer mitochondrial membranes of most organisms (De Pinto et al., 2003). The OMM functions as a link between mitochondrial metabolism and the rest of the cell (Cassara et al., 2009). Some other reports also demonstrated the presence of VDACs in the plasma membrane and other organelles (Bathori et al., 2000; Shoshan-Barmatz et al., 2006). *In vitro* transcription of *VDAC* was constructed to observe the distribution of VDAC in mouse oocytes and to confirm the relationship between [Ca^2+^]_m_ and VDAC. The distribution of [Ca^2+^]_m_ and VDAC were obviously different. VDAC distributed in most parts of oocytes (Supplementary Figure S3).

### Calmodulin and Higher [Ca^2+^]_m_ Mitochondria Distribution in Oocytes

Calmodulins (CaMs), including two members termed CaM1 and CaM2, are thought to be the initial responding proteins in the cell to sense dynamic changes in Ca^2+^ concentration. CaMs are expected to distribute in oocytes around the most sensitive organelles for Ca^2+^. Firstly, we cloned the CaM1 and CaM2 cDNA sequences and linked them with mCherry as pGEMHE-CaM1-mCherry and pGEMHE-CaM2-mCherry plasmids (Supplementary Figure S1); then we observed the localization of CaMs during oocyte maturation. Both CaM1 and CaM2 are located at the spindle (Supplementary Figure S4), which indicates that CaM1 and CaM2 have similar locations and functions in spindle formation as previously reported (Xu et al., 1996; Mehta et al., 2014).

**FIGURE 7:**
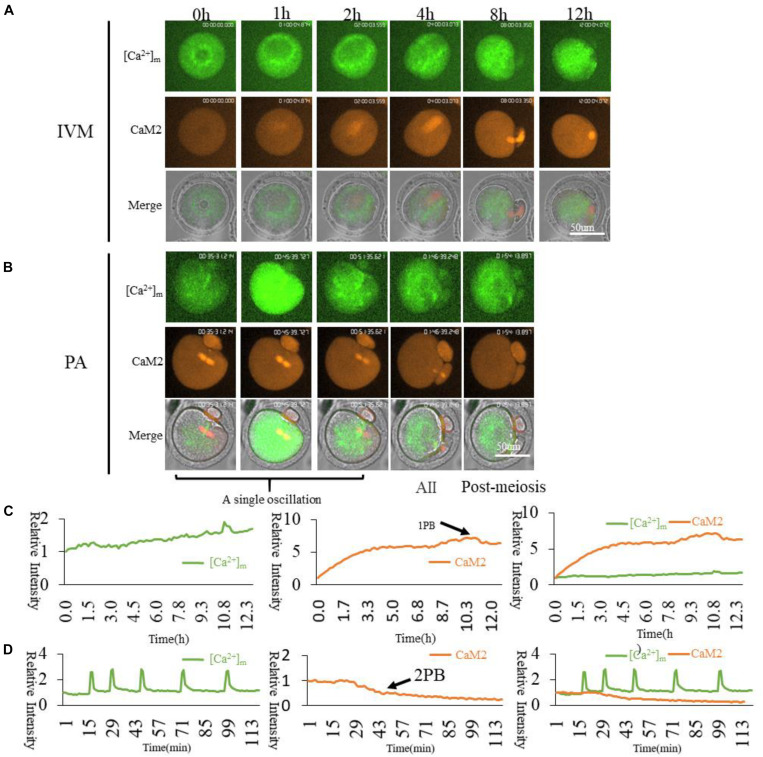
CaM2 and higher [Ca^2+^]_m_ mitochondria dynamic distribution during oocyte maturation and activation. **(A,B)** CaM2 and higher [Ca^2+^]_m_ mitochondria distribution during oocyte maturation and activation. IVM, *In vitro* Maturation; PA, Parthenogenetic Activation. **(C,D)** Fluorescence intensity of CaM2 (Red) and [Ca^2+^]_m_ (Green) changes during maturation and activation. Ordinate is marked as Relative Fluorescence Intensity (relative to the fluorescence intensity of the start point).

Since CaM1 and CaM2 have similar distributions, we wanted to confirm the relationship between [Ca^2+^]_m_ and CaM2. Higher [Ca^2+^]_m_ mitochondria surrounded the spindle, while CaM2 was localized on the spindle (Figure 7). We conclude that CaM2 is more directly involved in spindle formation, while [Ca^2+^]_m_ regulates mitochondrial activity for energy supplementation. [Ca^2+^]_i_ oscillations triggering cytoplasmic Ca^2+^ increase during oocyte activation will synchronize mitochondrial energy supplementation, spindle formation and maintenance as well as other cellular events during oocyte activation.

## Discussion

Abnormal metabolism such as hypertension, hyperglycemia and hyperlipidemia will cause long-term stress in the oocyte’s endoplasmic reticulum and mitochondria (Wang et al., 2019), which severely damages the quality of oocytes, thereby leading to lower pregnancy rates in women (Jungheim et al., 2013a). Increased Ca^2+^ signaling plays an important role in oocyte activation, which is a prerequisite for embryonic development (Dupont et al., 2010). Ca^2+^ signaling to specific organelles, such as the mitochondrial matrix for oxidative metabolism (Griffiths and Rutter, 2009), regulates organelle-specific cell functions (Marchant et al., 2002). Mitochondrial damage in oocytes reduces a woman’s reproductive capacity (Jungheim et al., 2013b), caused by inefficient oocyte activation (Ou and Sun, 2017).

Mitochondrial Ca^2+^ uptake has been studied for more than five decades (Rottenberg and Scarpa, 1974). Mitochondrial matrix calcium ([Ca^2+^]_m_) dynamics plays vital roles in regulating fundamental cellular and organelle functions including bioenergetics (Van Blerkom et al., 1995; Dumollard et al., 2007; Fink et al., 2017). The cytosolic Ca^2+^ influx to mitochondria (Viola et al., 2009) increases [Ca^2+^]_m_ to stimulate important enzymes in the Krebs cycle (Rizzuto et al., 2012), which provides enough ATP to support the increased energy requirement for maintaining mammalian oocyte activation (Chacko and Eliceiri, 2018). A large amount of Ca^2+^ is accumulated in the mitochondrial matrix to promote the transition of mitochondria from the resting state to the activated state (Dumollard et al., 2006), and changes in [Ca^2+^]_m_ will modify the mitochondrial metabolism to a certain extent. It is known that mitochondrial Ca^2+^ uptake is the basis for providing the necessary link between ATP supply and demand during cardiomyocyte contraction (Hajnoczky et al., 1995). Disturbances in Ca^2+^ fluxes lead to reduced bioenergetics of the cell or cellular death (Naon and Scorrano, 2014; Mishra, 2016; Samanta et al., 2018). In contrast, severe heart disease, such as reperfusion injury, is caused by excessive intake of mitochondria to induce Ca^2+^ overload and increase ROS production, even leading to cell apoptosis (Gordo et al., 2002; Ozil et al., 2005; Bonora et al., 2017). To prevent this, elevated [Ca^2+^]_m_ rapidly migrates from the mitochondria into the cytoplasm and ER (Hajnoczky et al., 1995; Berridge et al., 2000; Bootman et al., 2001) which is essential for cells to perform accurate functions. Our previous study showed that mitochondrial activity plays an important role in the maintenance of [Ca^2+^]_i_ oscillations during oocyte activation (Wang et al., 2018); moreover, [Ca^2+^]_i_ oscillations also activate mitochondrial ATP production during fertilization (Campbell and Swann, 2006). We focused on the interplay between mitochondrial metabolism and Ca^2+^ signaling during oocyte maturation and activation in this study.

Our current understanding of [Ca^2+^]_m_ regulation in oocytes is still limited. In order to determine the relationship between [Ca^2+^]_m_ and [Ca^2+^]_i_ dynamics, we focused on whether the Ca^2+^ changes in the mitochondria are independent or accompanied by changes in cytoplasmic [Ca^2+^]_i_ oscillations. Rhod2 is a dye used to detect mitochondrial Ca^2+^ (Dubouskaya et al., 2018). The localizations of Fluo-4 and Rhod2 in oocytes were significantly different. It was clearly seen that Fluo-4 was distributed in the cytoplasm (arrow in Figure 1A), while Rhod2 distribution was similar to that of the mitochondria (arrowhead in Figure 1A). From the fluorescence analysis results (Figure 1B), it can be seen that Fluo-4 AM and Rhod2 AM have the same patterns as oscillations. We thus have confirmed that Ca^2+^ in the cytoplasm and in the mitochondria change simultaneously.

Although Rhod2 is used as a classic Ca^2+^ probe in mitochondria (Dubouskaya et al., 2018), it is not widely used in oocytes. In order to ensure an accurate pattern of the mitochondrial Ca^2+^ dynamic change, synchronized with the cytoplasmic [Ca^2+^]_i_ oscillations, we applied an encoded Ca^2+^ probe with N-terminal mitochondrial localization signal peptide (Mt-GCaMP6s) to detect the patterns of [Ca^2+^]_m_ (Supplementary Figure S1A). After injection of Mt-GCaMP6s cRNA, [Ca^2+^]_m_ still exhibited oscillation patterns synchronized with Rhod2. Because Rhod2 displayed exactly the same pattern as [Ca^2+^]_i_ oscillations (Figure 1), [Ca^2+^]_m_ exhibited a same pattern in synchrony with [Ca^2+^]_i_. We also found that there are some differences in the position of Rhod2 and Mt-GCaMP6s; Rhod2-stained mitochondria are increased in quantity compared to Mt-GCaMP6s positive mitochondria. Indeed, most mitochondria can be stained by Rhod2, while only some of them displaying much higher [Ca^2+^]_m_ mitochondria will be stained by Mt-GCaMP6s (Figure 2). Comparative consideration of such two mitochondrial Ca^2+^ dyes, Mt-GCaMP6s may be more suitable to distinguish the changes of Ca^2+^ concentration in mitochondria. Further studies are needed to better understand the difference between Rhod2 and Mt-GCaMP6s positive mitochondria.

Next, we wanted to detect the dynamic changes of [Ca^2+^]_m_ during GV oocyte maturation. We found that higher [Ca^2+^]_m_ mitochondria distributed around the germinal vesicle (Figure 3 and Supplementary Video S1). After GVBD, [Ca^2+^]_m_ was higher in the area surrounding the spindle. During emission of the first polar body, [Ca^2+^]_m_ in the equatorial plate area increased significantly. Mitochondrial metabolism is much stronger in the energy-demanding part of the oocyte. Interestingly, there were two spikes of [Ca^2+^]_m_ around the time of first polar body extrusion (Figure 3B). Right curve indicates “GVBD but 1st poly body- non-extruded oocytes” displayed that maturation failed oocytes after GVBD, which lacks a [Ca^2+^]_m_ dynamic changes pattern as that matured MII oocytes. The irradiation of the laser caused failure of the first polar body extrusion in some oocytes (Figure 3C). The 2nd spike only displayed in second meiotic oocytes which had extruded 1st polar body. The highest [Ca^2+^]_m_ was found in MII oocytes in preparation for activation. These two time points are likely the most energy requiring critical periods for oocytes.

Is this dynamic change in mitochondrial [Ca^2+^]_m_ related to activated mitochondria? To address this question the relationship between [Ca^2+^]_m_ and the distribution of active mitochondria was studied. MitoRed is a dye localized to activated mitochondria (Chazotte, 2011). During GV oocyte *in vitro* maturation, we observed [Ca^2+^]_m_ by Mt-GCaMP6s and activated mitochondria by MitoRed (Figure 4), respectively. Mito-Red may have cytotoxicity for long-term observations, so that we could not obtain a clear imaging as Mt-GCaMP6s single signal (488 nm excitation) during oocyte maturation (Figure 3A). In order to obtain a clear location information of [Ca^2+^]_m_ and activated mitochondria, we performed a short 4-h observation with double concentration of MitoRed (Supplementary Figure S2). Double concentration of Mito-Red led to overexposure and cytotoxic effects after more than 4 h of observation. Higher [Ca^2+^]_m_ and activated mitochondria showed a much clearer co-localization than during long term observation. If higher [Ca^2+^]_m_ was co-localized with activated mitochondria, Ca^2+^ dynamic entry into mitochondria may participate in mitochondrial metabolism transfer from the resting to the activated state. There was a clear co-localization between higher [Ca^2+^]_m_ and MitoRed-positive mitochondria surrounding the chromosomes (Figure 4 and Supplementary Figure S2). During GV oocyte maturation from prophase I to metaphase II, chromosomes and the spindle require energy in the entire oocyte. The co-localization of higher [Ca^2+^]_m_ and MitoRed-positive mitochondria indicated that higher activated mitochondria required a higher [Ca^2+^]_m_. The localization relationship between [Ca^2+^]_m_ and active mitochondria during oocyte partheno-activation was also observed next (Figure 5). [Ca^2+^]_m_ and MitoRed still had clear co-localizations in the resting state. With [Ca^2+^]_i_ oscillations, all of the mitochondria in the entire oocyte have a higher [Ca^2+^]_m_, increasing in the flashing state, which was consistent with the result of Figure 1. While oocytes were in the cytoplasmic Ca^2+^ flashing state, the [Ca^2+^]_m_ increases, and the MitoRed positive mitochondria remain in a stable distribution pattern. One part of the mitochondria maintained a higher [Ca^2+^]_m_ around the nucleus, and the other mitochondrial [Ca^2+^]_m_ will increase instantaneously carried by [Ca^2+^]_i_, resulting in a pulse-like function enhancement. The role of [Ca^2+^]_m_ oscillations on mitochondrial metabolism needs to be further studied.

The MCU, which is identified as a highly selective Ca^2+^ channel (Xing et al., 2019), uptakes Ca^2+^ across the inner mitochondrial membrane, playing critical roles in various mitochondrial functions (Kirichok et al., 2004; Mammucari et al., 2017). MCU consists of multiple subunits, and its Ca^2+^ influx activity is controlled by regulatory subunits (Yu et al., 2014), including two regulators MICU1 (Perocchi et al., 2010) and MICU2 (Patron et al., 2014). MICU1is localized to the inner mitochondrial membrane with conformational changes upon Ca^2+^ binding (Kamer and Mootha, 2014). In our study, MICU distributed with higher [Ca^2+^]m mitochondria in the GV oocytes (Figure 6), however in PAoocytes, distribution of MICU was similar as activated mitochondria with no change as [Ca^2+^]_i_ oscillations. This may indicate that MICU is related to mitochondrial Ca^2+^ and mitochondrial activity. We also observed that VDAC (Supplementary Figure S4), which is widely believed to be distributed on the outer membrane, has a different distribution from the higher [Ca^2+^]_m_ mitochondria, indicating that the process of regulating Ca^2+^ entry and exit from the mitochondrial matrix into and out of the mitochondria is not related to VDAC.

Dynamic Ca^2+^ responds to its corresponding functions, and this change is determined by a protein initiation signal pathway with [Ca^2+^]_i_ sensing. Most studied Ca^2+^-responsive proteins are members of the calmodulin family. The calmodulin family contains CaM1 and CaM2. We examined the relationship between CaMs and higher [Ca^2+^]_m_ of mitochondria. We cloned CaM1 and CaM2 linked with mCherry (Supplementary Figure S1). In GV oocytes, two types of cRNA were injected and time-lapse confocal laser microscopy was conducted. We found both CaM1 and CaM2 in the spindle area (Supplementary Figure S4). CaM2 and [Ca^2+^]_m_ were examined to show the relationship between cytoplasmic Ca^2+^sensor CaM2 and active mitochondria. It can be seen in Figure 7 that higher [Ca^2+^]_m_ mitochondria are distributed surrounding the spindle, however, CaM2 is associated with the spindle (Figure 7A). When activated, CaM2 associates with the spindle until the second polar body extrusion (Figure 7B). The main function of the CaMs family may be directly involved in microtubule organization and spindle formation as described before (Fan and Sun, 2004). The distribution of higher [Ca^2+^]_m_ and activated mitochondria around the spindle also indicates that the change of calcium-regulated spindle dynamic changes requires a continuous supply of energy by higher [Ca^2+^]_m_ stimulated mitochondria. Cortical region Mito-Red signals are independent of [Ca^2+^]_m_ dynamic changes, which was not our expected result. Cortical [Ca^2+^]_m_ oscillations may have some other function of mitochondria regulations. There may be another possibility that another probe but not Mito-Red is suitable for detecting the mitochondrial state during partheno-activation. An accurate distribution of higher [Ca^2+^]_m_ mitochondria in oocytes is important for oocyte spindle reformation.

Deepening our understanding and basic knowledge about oocyte maturation/activation and energy metabolism is of great value for solving clinical problems such as insufficient oocyte cytoplasmic maturation and activation as well as insufficient energy supply caused by mitochondrial damage. Studying the regulatory mechanisms between [Ca^2+^]_m_ and mitochondrial metabolism in oocytes will provide more possibilities for improving the efficiency of assisted reproduction.

## Data Availability Statement

The raw data supporting the conclusions of this article will be made available by the authors, without undue reservation.

## Ethics Statement

All mice were handled in accordance with the institutional animal care policies of the Institute of Zoology, Chinese Academy of Sciences. The Laboratory Animal Care and Use Committee of the Institute of Zoology approved this study.

## Author Contributions

FW, Q-YS, and X-HO conceived and designed the experiments. FW and others conducted experiments. FW and Q-YS analyzed the data. FW, HS, X-HO, and Q-YS wrote the manuscript. All authors contributed to the article and approved the submitted version.

## Conflict of Interest

The authors declare that the research was conducted in the absence of any commercial or financial relationships that could be construed as a potential conflict of interest.

## Supplementary Material

Supplementary Figure 1 Constructed vectors for *in vitro* expression. **(A,B)** Complete cyclic plasmids maps of Mt-GCaMP6s and mCherry labeled protein. **(C–F)** Templates for cRNA *in vitro* expression by PCR with primers M13F and M13R.

Supplementary Figure 2 Short term observation of higher [Ca^2+^]_m_ and activated mitochondrial distribution during oocyte maturation. Double concentration of Mito-Red led to overexposure and cytotoxic effect after more than 4 h of observation. Higher [Ca^2+^]_m_ and activated mitochondria show clear co-localization.

Supplementary Figure 3 Dynamic distribution of VDAC and higher [Ca^2+^]_m_ mitochondria during oocyte maturation and activation. **(A,B)** VDAC and higher [Ca^2+^]_m_ mitochondria distribution during oocyte maturation and activation. C: Fluorescence intensity of VDAC (Red) and [Ca^2+^]_m_ (Green) changes during maturation and activation. Ordinate is marked as Relative Fluorescence Intensity (relative to the fluorescence intensity of the start point).

Supplementary Figure 4 CaM1 and CaM2 distribution in mature oocytes. Both CaM1 and CaM2 are located at the spindle.

Supplementary Video 1 [Ca^2+^]_m_ dynamic changes during oocyte maturation.

## Abbreviations

[Ca^2+^] I: Intracellular Ca^2+^ Concentration
no[Ca^2+^]m: Mitochondrial Matrix Ca^2+^ Concentration
cpGFP: Circularly Permuted Green Fluorescent Protein
GV: Germinal Vesicle
GVBD: Germinal Vesicle Breakdown
MII: Metaphase II
Mt-GCaMP6s: Encoding Mitochondrial Ca^2+^ Probe Based cpGFP With A Mitochondrial Signal Peptide.
