# Supplementary material for: Mitochondrial Ca2 + Is Related to Mitochondrial Activity and Dynamic Events in Mouse Oocytes
Source: Front Cell Dev Biol. 2020 Oct 27;8:585932. doi: 10.3389/fcell.2020.585932 (PMC7652752; doi:10.3389/fcell.2020.585932)
Supplement: Supplementary Figure 1 — Constructed vectors for in vitro expression. (A,B) Complete cyclic plasmids maps of Mt-GCaMP6s and mCherry labeled protein. (C–F) Templates for cRNA in vitro expression by PCR with primers M13F and M13R. [file Image_1.pdf]

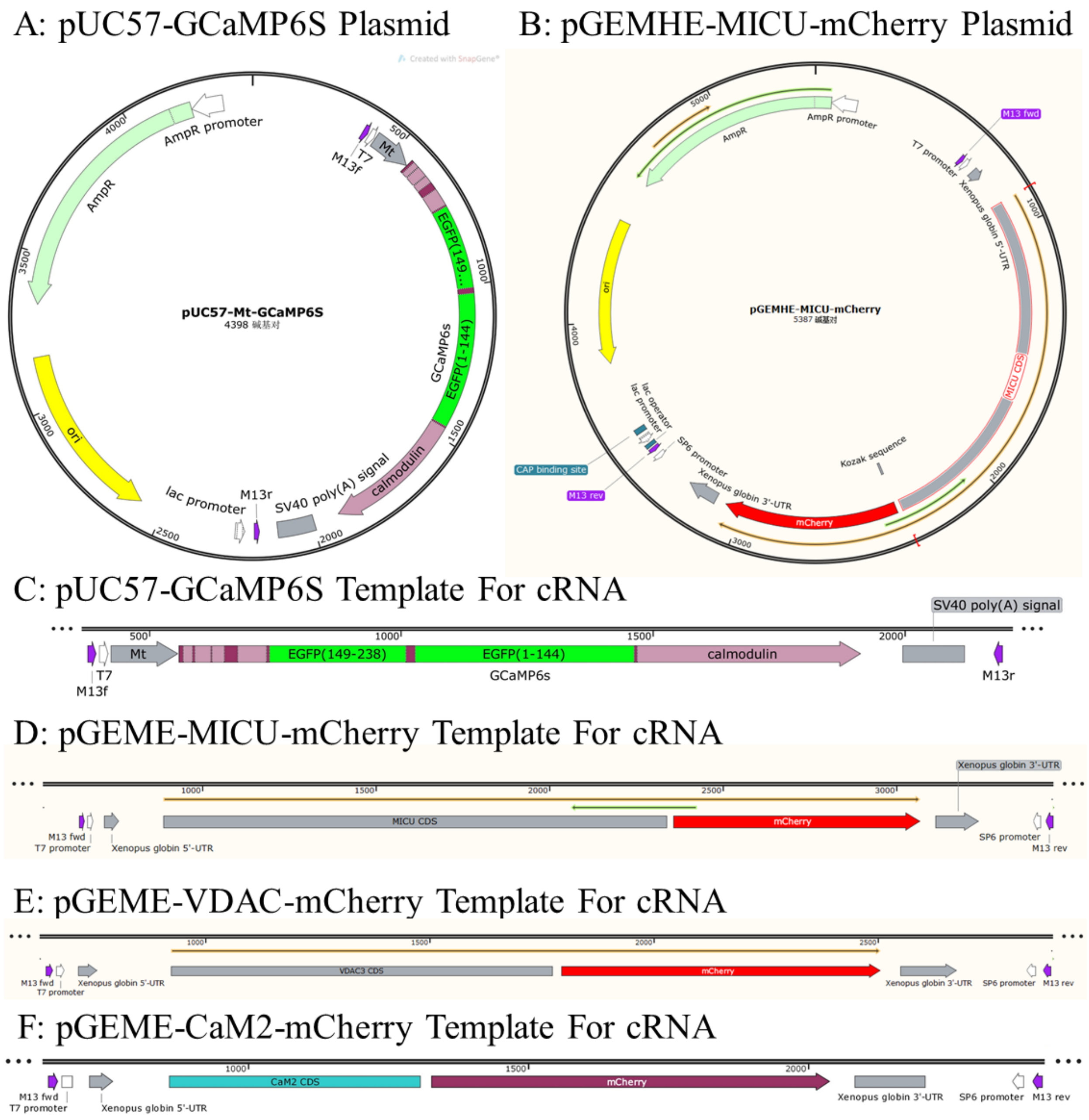

Supplementary Figure 1. Constructed vectors for in vitro expression. A and B: Complete cyclic plasmids maps of Mt-GCaMP6s and mCherry labeled protein. C toF: Templates for cRNA in vitro expression by PCR with primers M13F and M13R.

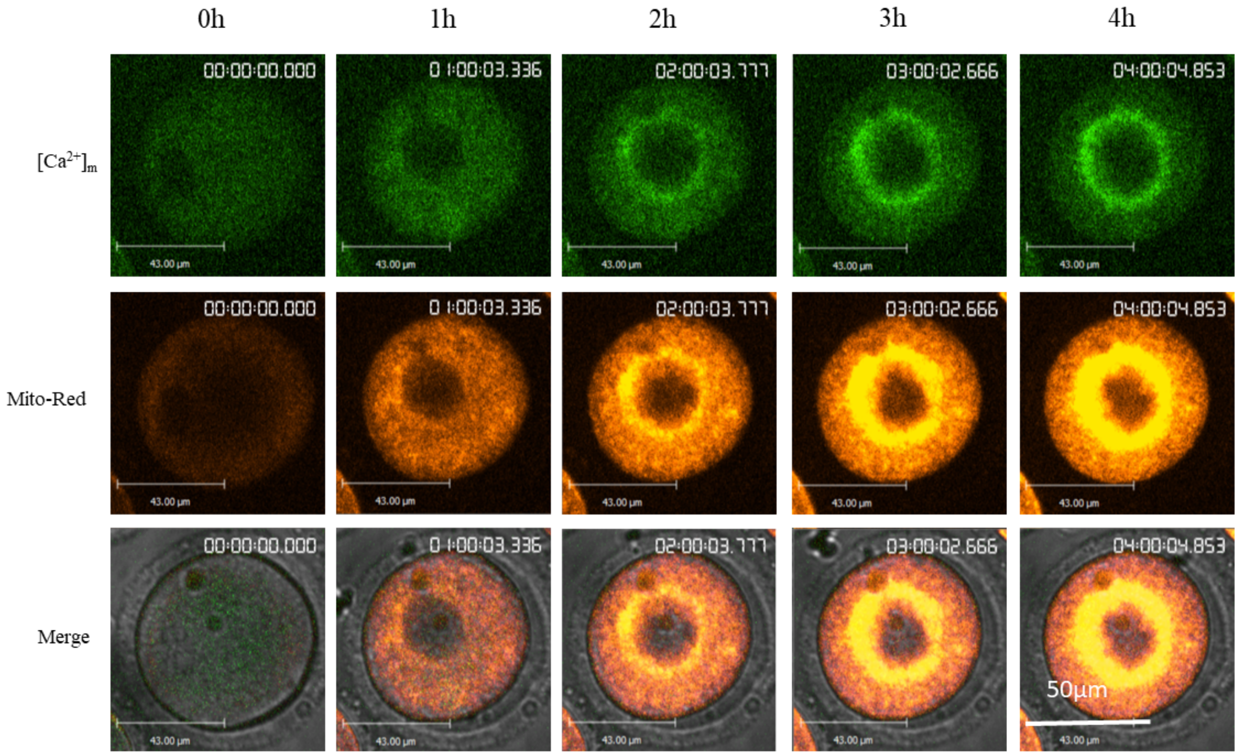

Supplementary Figure 2. Short term observation of higher $[Ca^{2+}]_m$ and activated mitochondrial distribution during oocyte maturation. Double concentration of Mito-Red led to overexposure and cytotoxic effect after more than 4 hours of observation. Higher $[Ca^{2+}]_m$ and activated mitochondria show clear co-localization.

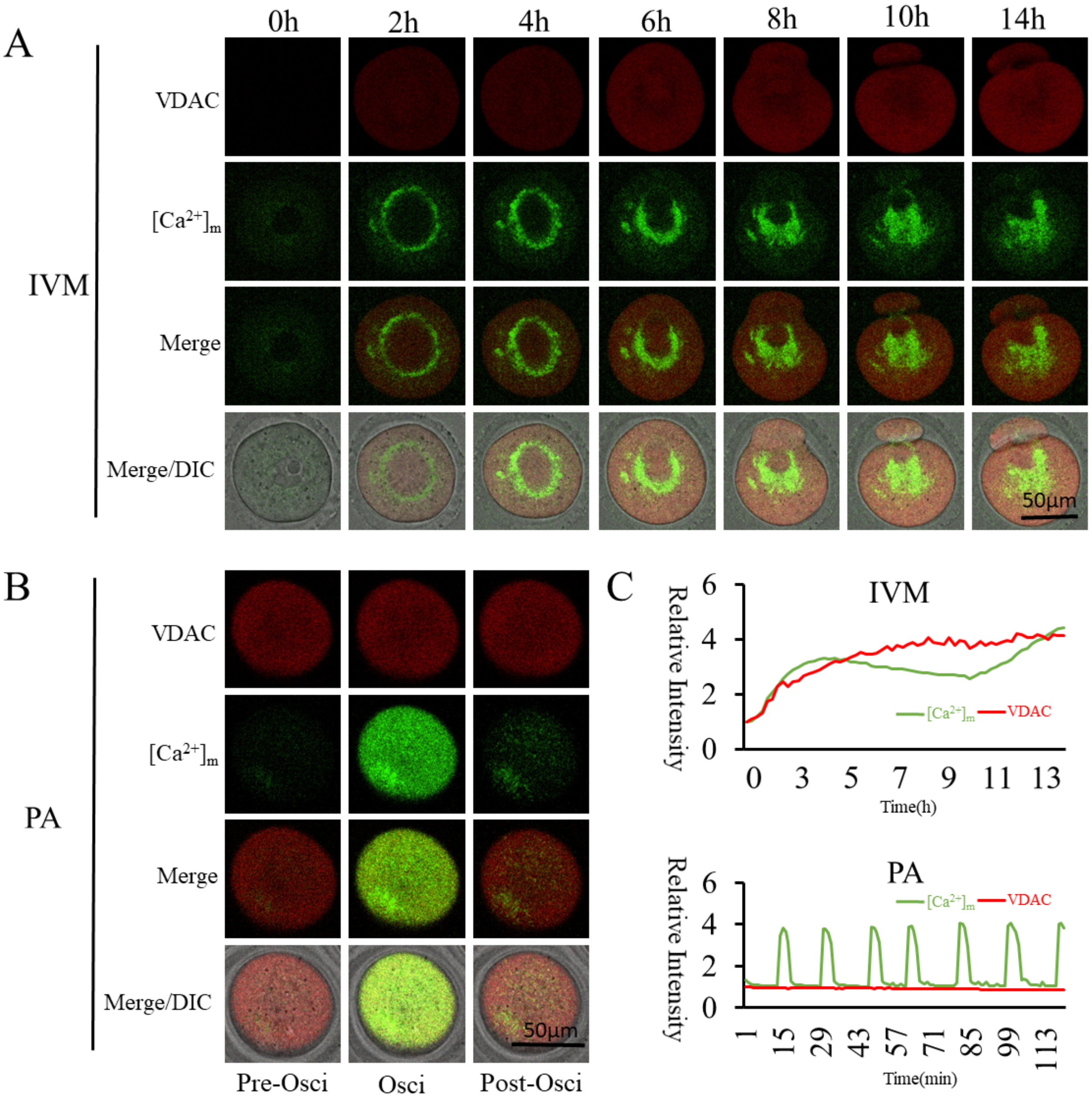

Supplementary Figure 3. Dynamic distribution of VDAC and higher $[Ca^{2+}]_m$ mitochondria during oocyte maturation and activation. A and B: VDAC and higher $[Ca^{2+}]_m$ mitochondria distribution during oocyte maturation and activation. C: Fluorescence intensity of VDAC (Red) and $[Ca^{2+}]_m$ (Green) changes during maturation and activation. Ordinate is marked as Relative Fluorescence Intensity (relative to the fluorescence intensity of the start point).

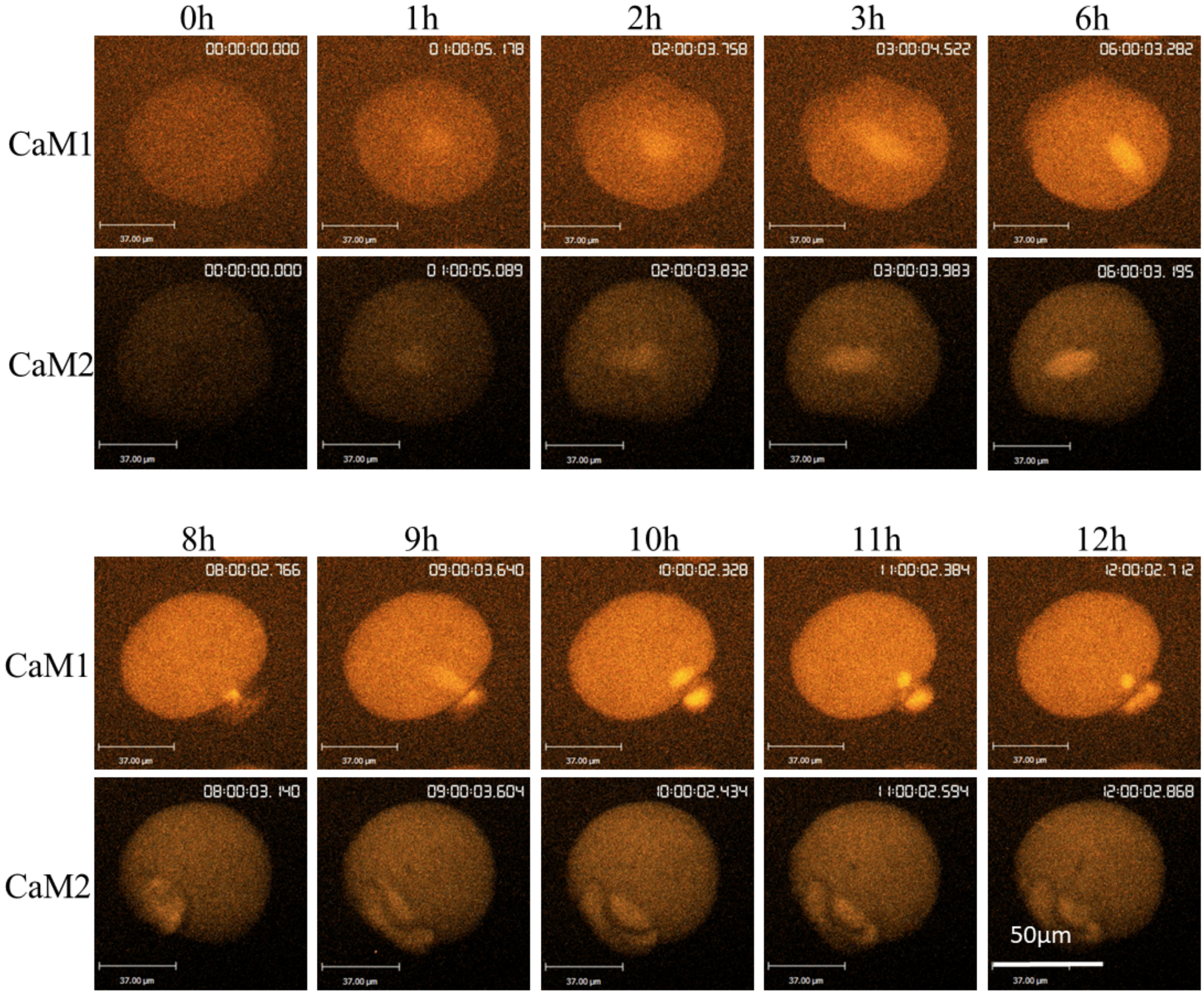

Supplementary Figure 4. CaM1 and CaM2 distribution in mature oocytes. Both CaM1 and CaM2 are located at the spindle.
